# Superior mesenteric artery syndrome complicated with duodenal bulb–descending ulcerative stricture: a case report and literature review

**DOI:** 10.3389/fmed.2026.1764870

**Published:** 2026-04-13

**Authors:** Erhua Yang, Jiajing Zhang, Zhimeng Yang, Suping Xiong, Fanming Guo, Dejiang Zhao, Xuefeng Wang

**Affiliations:** Nujiang People’s Hospital, Nujiang, China

**Keywords:** cicatricial ulcer, duodenal stasis syndrome, duodenal stenosis, gastrointestinal obstruction, superior mesenteric artery syndrome

## Abstract

**Background:**

Duodenal stasis syndrome is a clinical condition characterized by impaired emptying of the proximal duodenum, arising from either functional motility disorders or mechanical obstruction. Superior mesenteric artery syndrome (SMAS) is the most recognized mechanical cause; however, its coexistence with ulcer-related duodenal strictures is rare and presents considerable diagnostic and therapeutic challenges.

**Case presentation:**

A 40-year-old man presented with recurrent postprandial epigastric distension and vomiting for more than 30 years. Endoscopy revealed complex ulcers with stenosis involving the duodenal bulb and descending segment, and CT angiography demonstrated markedly reduced aortomesenteric angle (AMA) and distance (AMD), consistent with SMAS. Given the failure of long-term conservative therapy, laparoscopic side-to-side gastrojejunostomy with Braun anastomosis was performed, resulting in complete symptom resolution and favorable weight recovery at one-year follow-up.

**Discussion:**

This case illustrates how SMAS and ulcer-induced strictures may create a dual-level obstruction forming a vicious cycle, and emphasizes the need for integrated evaluation using CT angiography and high-resolution endoscopy. Therapeutic strategies remain debated among conservative, endoscopic, and surgical approaches, whereas minimally invasive bypass surgery offers more durable outcomes in patients with fixed fibrotic strictures.

**Conclusion:**

Clinicians should maintain a high index of suspicion for SMAS and associated lesions in patients with long-standing upper gastrointestinal obstruction. Complementary assessment using CT angiography and endoscopy is essential for identifying complex obstructive mechanisms. In selected patients with combined vascular compression and fixed duodenal stenosis, laparoscopic bypass reconstruction may represent an effective therapeutic option, although further evidence from larger clinical series is required to confirm its long-term efficacy.

## 1 Introduction

Duodenal stasis syndrome is a multifactorial upper gastrointestinal disorder characterized by a combination of functional and mechanical impairment, manifesting clinically as postprandial epigastric distension, nausea, vomiting, and delayed gastric emptying. Its etiologies are diverse and include duodenal dysmotility, ulcer-related cicatricial stenosis, and external compression—most notably compression by the superior mesenteric artery.

Superior mesenteric artery syndrome (SMAS), also referred to as Wilkie’s syndrome, represents an uncommon but clinically significant cause of proximal intestinal obstruction. The disorder occurs when the third portion of the duodenum becomes compressed between the abdominal aorta and the superior mesenteric artery, leading to impaired passage of intestinal contents and varying degrees of gastric outlet obstruction (1), with reported incidence estimates ranging between approximately 0.1 and 0.3% in the general population (2, 3).

The most prominent feature of SMAS is the reduction of the aortic mesenteric angle (AMA) and the shortening of the aortic mesenteric distance (AMD), leading to compression of the third segment of the duodenum. In certain patients, SMAS may coexist with duodenal ulcer disease and subsequent scarring, transforming the disease from a single pathological process into a dual mechanism of “dynamic vascular compression plus fixed mechanical narrowing,” thereby markedly increasing the complexity of diagnosis and management.

Here, we report a rare and illustrative case of SMAS complicated by chronic ulcer-related stenosis involving the duodenal bulb and descending segment. To our knowledge, reports describing the coexistence of proximal ulcer-related fibrotic stenosis and SMA-induced vascular compression producing a dual-level obstruction are extremely limited. This case highlights the diagnostic challenges and therapeutic decision-making when two distinct obstructive mechanisms coexist.

## 2 Case presentation

### 2.1 Basic information

Patient: A 40-year-old man.
Chief complaint: Recurrent postprandial epigastric fullness, nausea, and vomiting for more than 30 years.

The patient reported that these symptoms initially occurred intermittently during adolescence and gradually increased in frequency over the following decades. During this period, no significant weight loss was documented until the last 2–3 years prior to admission, when progressive postprandial intolerance led to reduced oral intake and mild weight reduction (BMI 19.3 at presentation), which were more readily triggered by a regular diet. Symptom exacerbations were accompanied by dizziness and headache, without fever, chills, dyspnea, or diarrhea. Long-term treatment with domperidone and cimetidine, together with adherence to a liquid diet, provided only transient relief, with rapid relapse upon medication withdrawal or resumption of a regular diet. On admission, the patient appeared emaciated (BMI 19.3) and had no prior surgical history or known systemic diseases.

He denied a history of diabetes, hypertension, chronic gastrointestinal disease, or long-term use of nonsteroidal anti-inflammatory drugs. There was no history of smoking, alcohol abuse, or previous abdominal trauma.

### 2.2 Physical examination

He was alert and markedly underweight. The abdomen was soft, without guarding. Mild epigastric tenderness was noted, with a questionably positive succussion splash.

## 3 Investigations

### 3.1 Laboratory tests

Complete blood count, liver and renal function tests, serum electrolytes, as well as amylase and lipase levels were all within normal limits. *Helicobacter pylori* infection status was assessed using a urea breath test, and the result was negative. The patient had been receiving long-term acid-suppressive therapy, which may influence serum gastrin levels. Moreover, acid-suppressive medication was not discontinued during the current hospitalization, which may therefore render serum gastrin measurements unreliable. Given that both imaging and endoscopic examinations clearly demonstrated a mechanical obstruction, a formal gastric emptying study was not performed.

### 3.2 Endoscopic findings

Upper gastrointestinal endoscopy demonstrated the presence of an A1-stage compound ulcer according to the Sakita–Miwa classification (active stage), a system commonly used in East Asian clinical practice at the pyloric canal–bulbar–descending junction. The patient also exhibited chronic superficial gastritis with mucosal erosions, accompanied by bile reflux. Examination of the duodenum revealed bulbitis with marked luminal deformity and stenosis. Targeted biopsies were obtained from the stenotic region at the bulb–descending junction to exclude malignant or other specific etiologies. Histopathological examination revealed chronic inflammatory changes without evidence of dysplasia or malignancy. The Sakita–Miwa classification was used to assess ulcer stage, whereas the Forrest classification is more commonly applied in Western practice.

### 3.3 CT angiography

**Figure 1:**
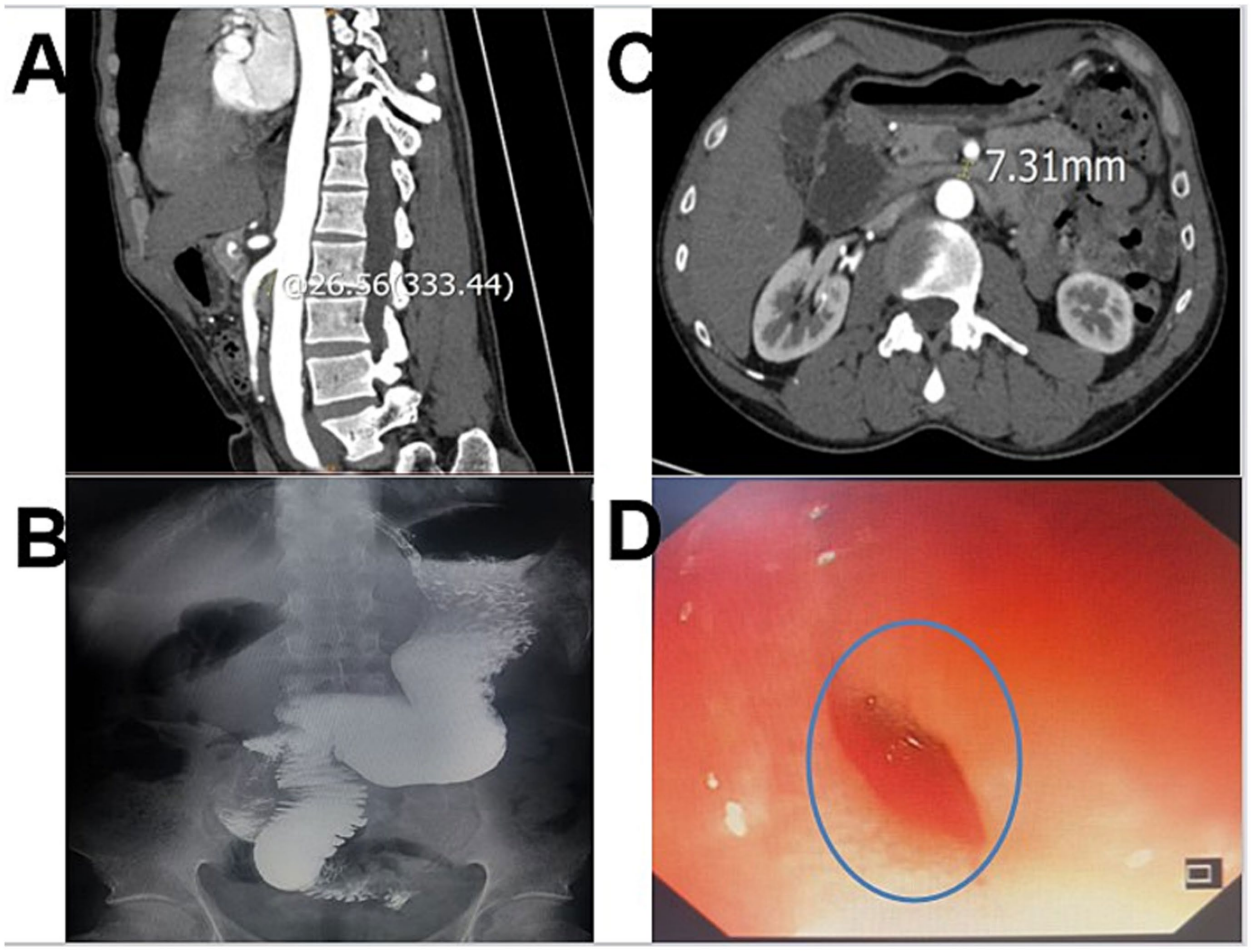
**(A)** Sagittal CT image demonstrating compression of the duodenum, consistent with SMAS. **(B)** Upper gastrointestinal barium study revealing duodenal obstruction. **(C)** Traverse CT image showing duodenal compression, further supporting the diagnosis of SMAS. **(D)** Gastroscopy revealed a duodenal ulcer with concomitant stenosis.

CT angiography revealed a significantly reduced aortomesenteric angle (AMA) and a shortened aortomesenteric distance (AMD), the measured aortomesenteric angle was 26.56°, and the aortomesenteric distance was approximately 7.31 mm, accompanied by compression and narrowing of the third portion of the duodenum. The bulbar–descending segment appeared dilated with fluid accumulation. No intra-abdominal mass or adhesive disease was detected. These findings were consistent with SMAS with associated stenosis of the duodenal bulbar–descending segment (see Figure 1).

## 4 Diagnosis

Based on the integrated findings from endoscopy and CT angiography, the final diagnosis was superior mesenteric artery syndrome accompanied by ulcer-related cicatricial stenosis of the bulbar–descending segment of the duodenum. Given the long clinical history and the presence of fibrotic ulcer-related stenosis, the chronic ulcer disease was regarded as the primary pathological process. The reduced aortomesenteric angle observed on CT angiography may therefore represent a contributing or aggravating factor rather than the sole primary etiology of obstruction.

## 5 Treatment

Following multidisciplinary team (MDT) discussion involving gastroenterology, surgery, radiology, nutrition, and endoscopy, surgical intervention was recommended owing to long-standing failure of conservative therapy and the presence of fixed cicatricial stenosis.

Duodenojejunostomy, which is commonly regarded as the standard surgical procedure for isolated SMAS, was carefully considered. However, endoscopic and imaging evaluation revealed severe fibrotic stenosis involving the duodenal bulb and descending segment. In this situation, duodenojejunostomy alone might not sufficiently bypass the proximal fixed obstruction. Endoscopic balloon dilation was also discussed; nevertheless, the stenosis appeared long-segment and fibrotic with marked luminal deformity, suggesting a high likelihood of recurrence and a potential risk of perforation with repeated dilation. Endoscopic gastroenterostomy was also considered; however, it was not selected because of limited long-term evidence and limited local availability. Therefore, laparoscopic side-to-side gastrojejunostomy with Braun anastomosis was selected to achieve reliable proximal bypass while reducing bile reflux.

## 6 Outcomes and follow-up

The patient passed flatus on postoperative day 3 and resumed a liquid diet. He advanced to a semiliquid diet on day 7 without vomiting. Upper gastrointestinal contrast study on postoperative day 9 showed normal gastric peristalsis, adequate filling of the duodenal bulb and descending segment, unobstructed passage through the horizontal and ascending portions, and smooth transit of contrast through the anastomosis into the jejunum. At 12-month follow-up, postprandial fullness and vomiting had completely resolved, and body weight increased from 61 kg to 70 kg (BMI 22.1). Although this follow-up period demonstrated satisfactory short-term symptom relief and nutritional recovery, longer observation will be necessary to evaluate the durability of the reconstruction, potential bile reflux, and late complications such as marginal ulceration.

## 7 Discussion

Because of the nonspecific clinical manifestations of SMAS and the overlap of symptoms with other gastrointestinal disorders, the condition is frequently underdiagnosed or diagnosed only after a prolonged period of investigation (4, 5). The present case illustrates a rare form of dual-level duodenal obstruction resulting from the coexistence of superior mesenteric artery compression and chronic ulcer-related cicatricial stenosis. While SMAS alone is an uncommon cause of proximal intestinal obstruction, its combination with fibrotic duodenal narrowing caused by long-standing peptic ulcer disease has been only rarely described. The coexistence of these two pathological mechanisms may create a complex obstructive process in which fixed luminal narrowing and vascular compression mutually aggravate duodenal stasis.

Although SMAS is rare, its coexistence with ulcer-induced duodenal stenosis has been reported only sporadically in the literature, mostly as isolated case reports. Currently, no reliable epidemiological data are available to quantify the incidence of this dual-etiology condition.

From an anatomical and pathophysiological perspective, the development of SMAS is closely related to the spatial relationship between the abdominal aorta and the superior mesenteric artery. Under normal conditions, the superior mesenteric artery arises from the anterior surface of the abdominal aorta and descends at an angle typically ranging from approximately 25° to 60°. Between these vascular structures lies a cushion of mesenteric fat and lymphatic tissue that maintains an adequate distance between the two vessels and protects the duodenum from compression (6). When this protective fat pad becomes depleted or when anatomical changes narrow the aortomesenteric angle, the duodenum may become trapped between the vessels, resulting in partial or complete obstruction. Imaging studies in patients with SMAS often demonstrate an aortomesenteric angle of less than 25° and an aortomesenteric distance of less than 8–10 mm, findings that are considered highly suggestive of the diagnosis (7).

SMAS is often associated with conditions that reduce the retroperitoneal fat pad, such as rapid weight loss, chronic illness, or severe nutritional depletion. However, adult presentations may be heterogeneous and sometimes occur in the absence of classical predisposing factors (8–10). These conditions can lead to significant loss of retroperitoneal adipose tissue, thereby reducing the aortomesenteric angle and predisposing the duodenum to vascular compression (10). However, more recent studies have suggested that the clinical spectrum of SMAS may be broader than previously appreciated. Adult patients with a variety of underlying conditions—including malignancy, postoperative weight loss, prolonged immobilization, or chronic gastrointestinal disease—may also develop this syndrome (1, 11). This expanding recognition highlights the importance of considering SMAS in the differential diagnosis of persistent upper gastrointestinal obstruction, even in patients without classic risk factors.

The clinical manifestations of SMAS are often variable and may evolve gradually over time. Patients frequently report postprandial abdominal pain, nausea, vomiting, early satiety, and progressive weight loss (4, 12). In many cases, symptoms are exacerbated after meals due to increased gastric and duodenal distension proximal to the site of compression (4, 13). Some patients also experience relief when assuming certain positions, such as lying in the left lateral decubitus or knee-chest position, which may transiently widen the aortomesenteric angle and reduce duodenal compression (4, 14, 15). Despite these characteristic features, the symptoms of SMAS are nonspecific and may mimic other gastrointestinal disorders, including functional dyspepsia, gastroparesis, chronic pancreatitis, or partial small bowel obstruction. As a result, delays in diagnosis are common, and many patients undergo extensive diagnostic evaluations before the underlying cause is identified (16).

Radiologic imaging plays a central role in establishing the diagnosis of SMAS. Computed tomography angiography has become one of the most widely used diagnostic modalities because it allows direct visualization of both vascular anatomy and duodenal morphology. Multiplanar reconstructions can accurately measure the aortomesenteric angle and distance while simultaneously demonstrating dilation of the proximal duodenum and stomach (17, 18). Ultrasonography has also been reported as a useful noninvasive technique for evaluating the aortomesenteric relationship, particularly in experienced hands (19, 20). Additionally, upper gastrointestinal contrast studies may reveal characteristic findings such as dilation of the first and second portions of the duodenum, abrupt compression at the third portion, and delayed transit of contrast material into the distal bowel (21).

Endoscopic evaluation, although not diagnostic for SMAS itself, plays an important complementary role in the diagnostic process. Gastroduodenoscopy allows clinicians to exclude other potential causes of duodenal obstruction, including intrinsic tumors, inflammatory strictures, or peptic ulcer disease (22). In some patients, endoscopy may reveal significant gastric retention or evidence of mucosal inflammation secondary to chronic stasis. In the present case, the combined findings from imaging and endoscopic examinations strongly suggested a mechanical obstruction related to vascular compression rather than a purely functional motility disorder.

The management of SMAS generally follows a stepwise approach beginning with conservative therapy (23, 24). Initial treatment strategies focus on nutritional rehabilitation, correction of fluid and electrolyte disturbances, and decompression of the stomach and proximal duodenum (25). Nutritional support is considered particularly important because restoration of body weight may increase the retroperitoneal fat pad and subsequently widen the aortomesenteric angle, thereby relieving the duodenal compression (26, 27). In some patients, positional therapy and enteral feeding through a nasojejunal tube may further facilitate symptom improvement. Reports in the literature indicate that conservative treatment may lead to symptom resolution in a subset of patients, especially when the condition is diagnosed early and when weight loss represents the primary underlying mechanism (28).

Despite these potential benefits, conservative management is not universally effective. Persistent symptoms, progressive malnutrition, or evidence of severe mechanical obstruction often necessitate surgical intervention (29). Several operative procedures have been described for the treatment of SMAS. Among these, duodenojejunostomy has become the most widely accepted surgical technique because it directly bypasses the compressed segment of the duodenum and restores normal intestinal continuity (30, 31). Advances in minimally invasive surgery have led to increasing adoption of laparoscopic duodenojejunostomy, which has demonstrated favorable outcomes in multiple case series, including high success rates and reduced postoperative recovery times (32, 33).

Alternative surgical options include Strong’s procedure, which involves division of the ligament of Treitz to mobilize the duodenum and reduce vascular compression (16), and gastrojejunostomy, which bypasses the obstructed duodenal segment by creating a connection between the stomach and jejunum (34). In the present case, surgical bypass reconstruction was ultimately selected after multidisciplinary evaluation because the patient had both vascular compression and fixed ulcer-related duodenal stenosis. The detailed rationale for the operative strategy has been described in the Treatment section. However, each technique has potential advantages and limitations, and the choice of procedure should be individualized based on the underlying anatomical abnormalities and clinical circumstances (34).

Another area of ongoing discussion in the literature concerns the optimal timing of surgical intervention. Some clinicians advocate prolonged conservative management in selected patients, whereas others emphasize earlier surgical treatment in cases with severe obstruction or persistent symptoms. The absence of large prospective studies has contributed to continued debate regarding the most appropriate treatment algorithm (30).

In the present case, the diagnosis of SMAS was supported by characteristic radiologic findings demonstrating narrowing of the aortomesenteric angle and compression of the third portion of the duodenum. Importantly, imaging and endoscopic examinations also confirmed the presence of mechanical obstruction, which helped differentiate the condition from functional disorders such as gastroparesis. This distinction is clinically relevant because the management strategies for these conditions differ substantially (4, 35).

Another consideration in this case involved the interpretation of laboratory findings related to gastric physiology. The patient had been receiving proton pump inhibitor therapy for an extended period prior to hospitalization, and the medication was not discontinued during the diagnostic evaluation. Proton pump inhibitors are known to influence serum gastrin levels by reducing gastric acid secretion and inducing compensatory hypergastrinemia. Consequently, gastrin measurements obtained during ongoing acid suppression therapy may not accurately reflect baseline physiological conditions (36, 37). In such circumstances, reliance on structural imaging findings becomes particularly important for identifying the underlying cause of obstruction.

The present case also underscores the diagnostic complexity of SMAS in adult patients. Compared with pediatric populations, adult cases are often associated with more heterogeneous clinical backgrounds and may occur without the classical precipitants of rapid weight loss or eating disorders (4). Some authors have even questioned whether SMAS represents a distinct pathological entity or rather a radiologic manifestation that may coexist with other gastrointestinal disorders (38). This debate highlights the importance of careful clinical correlation when interpreting imaging findings suggestive of vascular compression.

Although the short-term outcome in this patient was favorable, certain limitations should be acknowledged. As a single case report, the findings cannot be generalized to all patients with SMAS. Furthermore, long-term follow-up data are limited, which restricts the ability to evaluate potential late complications such as recurrent obstruction, marginal ulcer formation, or nutritional consequences. Future studies involving larger patient cohorts and longer follow-up periods will be necessary to better define optimal diagnostic and therapeutic strategies.

In summary, this case contributes to the growing body of literature describing the diverse clinical presentations of SMAS. The condition remains a diagnostic challenge due to its rarity and nonspecific symptoms. Early recognition requires a high degree of clinical suspicion and careful integration of clinical history, imaging findings, and endoscopic evaluation. Improved awareness of this entity among clinicians may facilitate earlier diagnosis and more appropriate management, ultimately improving outcomes for affected patients.

## 8 Limitation

This case report has several limitations. First, due to the continued use of PPI drugs prior to treatment, serum gastrin levels were not measured. Second, although endoscopy provided morphological evidence of stenosis, repeated biopsies from the stricture site were limited in number. Finally, as a single-case observation, the findings cannot establish a causal relationship between SMAS and ulcer-related stenosis.

## 9 Conclusion

This case highlights a rare presentation of dual-level duodenal obstruction caused by chronic ulcer-related stenosis complicated by superior mesenteric artery compression. In patients with long-standing symptoms of upper gastrointestinal obstruction, careful evaluation with complementary imaging and endoscopic studies is essential to identify coexisting pathological mechanisms. In the present case, chronic ulcer-induced duodenal stenosis likely represented the primary pathological process, whereas superior mesenteric artery compression may have acted as a contributing factor that aggravated the obstruction. Laparoscopic gastrojejunostomy with Braun anastomosis provided effective short-term symptom relief and nutritional improvement. However, given that this report describes a single case with limited follow-up, further studies and longer-term observations are required to determine the durability and broader applicability of this surgical approach.

## Data Availability

The original contributions presented in the study are included in the article/supplementary material, further inquiries can be directed to the corresponding author.
